# Occlusal height difference between maxillary central and lateral incisors: should aesthetic perception influence bracket placement?

**DOI:** 10.1186/s13005-019-0191-y

**Published:** 2019-02-26

**Authors:** Jan Hourfar, Dirk Bister, Björn Ludwig, Gero Kinzinger, Jörg Alexander Lisson

**Affiliations:** 1 Department of Orthodontics, Saarland University, Kirrberger Strasse 100, 66424 Homburg/Saar, Germany; 2 Department of Orthodontics, Guy’s and St Thomas’ NHS Foundation Trust, UK and King’s College Dental Institute, London, UK; 3 Private Practice Traben-Trarbach, Germany and Department of Orthodontics, Saarland University, Homburg/Saar, Germany; 4 Private Practice Tönisvorst, Germany and Department of Orthodontics, Saarland University, Homburg/Saar, Germany; 5 Department of Orthodontics, Saarland University, Homburg/Saar, Germany

## Abstract

**Background:**

The aim of this study was to verify anecdotal evidence that the maxillary central-to-lateral occlusal height difference (OHD) of more than 0.5 mm is a feature displayed in the majority of media and to discuss its implications for individualized orthodontic treatment planning.

**Methods:**

Photographs of smiling female models were collected from a variety of printed advertisements and allocated to 3 groups (*n* = 30 each): 1 dental, 2 fashion and 3 orthodontics. Group 4 used female patient images from orthodontic textbooks, assuming an OHD of 0.5 mm between maxillary central and lateral incisors. OHD was assessed by measuring the incisor height on the photographs and using average values to establish height differences.

**Results:**

The average maxillary central-to-lateral incisor OHD differences were 1.39 mm (dental literature), 1.34 mm (fashion advertisements), 1.23 mm (orthodontics) and 0.62 mm (orthodontic textbooks) respectively. The differences between the advertisement groups were not significant (*P* >  0.05), but for orthodontic textbooks they were (*P* <  0.001).

**Conclusions:**

Advertisers seem to prefer greater maxillary central-to-lateral OHD compared to commonly used bracket placement protocols. Therefore, discussing OHD at start of treatment is recommended; modification of commonly used bracket placement protocols may be helpful to achieve desired aesthetic outcome.

## Background

Features that contribute to an aesthetic smile have been well documented before [1–4]. The height difference between maxillary central and lateral incisors is one of them and most contemporary protocols for bracket placement prescribe a difference of 0.5 mm [5, 6].

However a number of authors [7–10], suggested that a maxillary central-to-lateral incisor occlusal height difference (OHD) of greater than 0.5 mm might improve aesthetics and anecdotal evidence from assessing smile lines displayed in advertisements appears to confirm this.

The aim of this study was to verify the maxillary central-to-lateral incisor OHD in females as a single feature in the aforementioned media and to discuss possible implications for individualized orthodontic treatment planning.

## Methods

### Photographic material

Over a two-year period, photographs of models with exposed gingival smile were collected from a variety of dental product catalogues and fashion magazines. To avoid selection bias, different groups (dental professionals and laypersons) were asked to contribute images. Dental professionals mainly contributed information from dental product advertising whereas laypersons collected fashion advertisements. The material was stored in boxes until assessment of the material for inclusion criteria was performed.

### Inclusion criteria

The material was assessed by two examiners for inclusion criteria. Based on visual assessment of the examiners, only female Caucasians [11, 12] with photographs taken in almost frontal view and with clearly identifiable incisal edges and gingival margins were included. To avoid specific bias, well-known models or celebrities were excluded [13]. A minimum required sample size (*n* = 27) was calculated based on a significance level of 0.05 and a power of 95% to detect a meaningful difference of 0.5 (±0.5 mm). More subjects than *n* = 27 were available for this investigation.

### Group allocation

The photographic material from the advertisements was allocated to three groups with *n* = 30 for each group:

- group 1 (dental): Advertisements for cosmetic dentistry products.
- group 2 (fashion): Advertisements for women’s fashion, designed for the general public.
- group 3 (orthodontic): Advertisements for orthodontic products.
- group 4 (orthodontic textbooks): differences in maxillary central-to-lateral incisor OHD were measured on post-treatment images of fixed appliance cases published in four orthodontic textbooks [14–17]. This group was used as a reference, assuming use of a protocol for bracket bonding prescribing a height difference of maxillary central-to-lateral incisors of 0.5 mm.

### Measurements

**Fig. 1:**
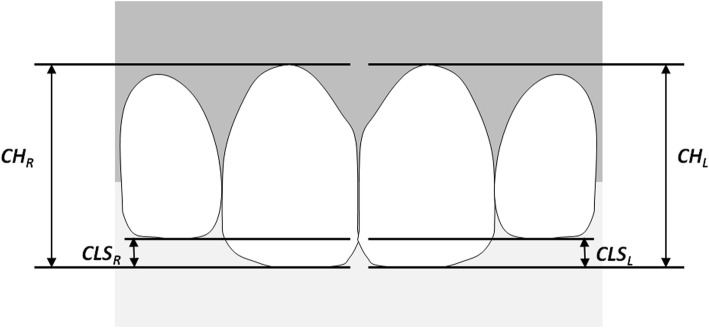
Definition of measurements. Crown height right and left side (CH_R_, CH_L_) of the central incisors; central-to-lateral incisal step right and left side (CLS_R_, CLS_L_)

All measurements on the photographic materials were performed manually using an orthodontic caliper (Münchner Modell®, Dentaurum, Ispringen, Germany) to a precision of 0.25 mm. The crown lengths of maxillary central incisors were measured from the incisal edge to the most apical point on the gingival margin. The maxillary central-to-lateral incisor steps were measured as the vertical distance between the incisal edges of maxillary central and lateral incisors (Fig. 1). Percentage of central-to-lateral incisal height difference for crown height of the ipsilateral tooth was calculated. The real crown lengths of the maxillary centrals were unknown and hence each calculated percentage was consecutively converted to a standardized value using a mean value of 10.5 mm (taken from the literature [2, 18]) for crown length of the maxillary central incisors. Using this method, a standardized value for each central-to-lateral OHD was calculated.

Calculating the real crown length using the standardized value of 10.5 mm, as described above, may have influenced our results. We therefore also evaluated results for different crown lengths and different maxillary central-to-lateral incisor height differences in 0.25 mm increments. Consecutively maxillary central-to-lateral incisor differences (Δ) were calculated using an average of 10.5 mm, 10 mm and 11 mm as base values for crown height of the maxillary central incisors, simulating a shorter or longer maxillary central incisor.

### Statistical analysis

Data was tabulated using Excel® (Microsoft Corp., Redmond, Washington, USA). In order to test intra-examiner reliability, the same operator repeated all measurements 3 months after initial measurements had been performed. Mean intra-examiner reliability (coefficient of variation; COV), was 0.08 (Range 0.03 to 0.11). The Kolmogorov-Smirnov-Test revealed normal distribution of the data therefore parametric analyses were undertaken. Differences between the groups were assessed using analysis of variance (ANOVA). Posthoc-Testing was performed using the Scheffé-Test. Descriptive statistics mean, standard deviation (sd), minimum (min) and maximum (max) are presented. The level of significance was set at 5%. SPSS™ for Windows®, version 22.0 (IBM Corp., Armonk, New York, USA) was used to perform the analyses.

## Results

Maxillary central-to-lateral incisor differences (Δ) calculated using an average of 10.5 mm, 10 mm and 11 mm as base values for crown height of the maxillary central incisors are presented in Table 1. These deviations increased for larger OHDs.

**Table 1:** Evaluation of different crown heights in relation to maxillary central-to-lateral incisor OHD

*Measured on photograph*		*Calculated maxillary central-to-lateral incisor difference based on average crown height of ...*					
Crown heightU1 (mm)	maxillary central-to-lateral incisor step (mm)	10.5 mm	10.0 mm	Δ (mm)	10.5 mm	11.0 mm	Δ (mm)
11	0	0.000	0.000	0.000	0.000	0.000	0.000
11	0.25	0.239	0.227	0.011	0.239	0.250	−0.011
11	0.5	0.477	0.455	0.023	0.477	0.500	−0.023
11	0.75	0.716	0.682	0.034	0.716	0.750	−0.034
11	1	0.955	0.909	0.045	0.955	1.000	−0.045
11	1.25	1.193	1.136	0.057	1.193	1.250	−0.057
11	1.5	1.432	1.364	0.068	1.432	1.500	−0.068
11	1.75	1.670	1.591	0.080	1.670	1.750	−0.080
11	2	1.909	1.818	0.091	1.909	2.000	−0.091
13	0	0.000	0.000	0.000	0.000	0.000	0.000
13	0.25	0.202	0.192	0.010	0.202	0.212	−0.010
13	0.5	0.404	0.385	0.019	0.404	0.423	−0.019
13	0.75	0.606	0.577	0.029	0.606	0.635	−0.029
13	1	0.808	0.769	0.038	0.808	0.846	−0.038
13	1.25	1.010	0.962	0.048	1.010	1.058	−0.048
13	1.5	1.212	1.154	0.058	1.212	1.269	−0.058
13	1.75	1.413	1.346	0.067	1.413	1.481	−0.067
13	2	1.615	1.538	0.077	1.615	1.692	−0.077
15	0	0.000	0.000	0.000	0.000	0.000	0.000
15	0.25	0.175	0.167	0.008	0.175	0.183	−0.008
15	0.5	0.350	0.333	0.017	0.350	0.367	−0.017
15	0.75	0.525	0.500	0.025	0.525	0.550	−0.025
15	1	0.700	0.667	0.033	0.700	0.733	−0.033
15	1.25	0.875	0.833	0.042	0.875	0.917	−0.042
15	1.5	1.050	1.000	0.050	1.050	1.100	−0.050
15	1.75	1.225	1.167	0.058	1.225	1.283	−0.058
15	2	1.400	1.333	0.067	1.400	1.467	−0.067

U1, Maxillary central(s); OHD, Occlusal height difference; Δ, Difference

**Fig. 2:**
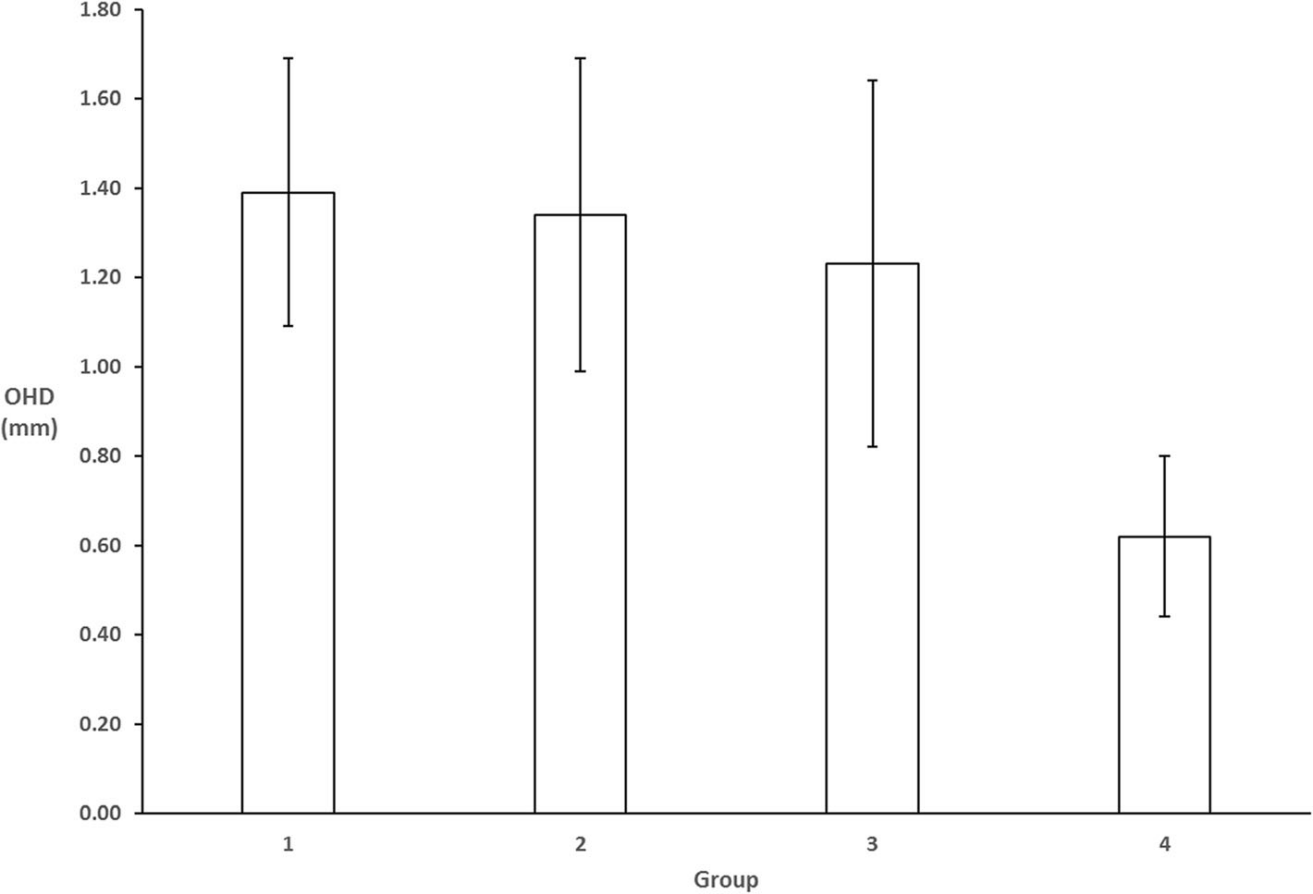
Calculated OHD based on results from measurements on photographs for the different groups

The results for the different groups are in Table 2 and Fig. 2. The average maxillary central-to-lateral OHD was more than 1 mm for all groups: 1 (dental), 2 (fashion) and 3 (orthodontics). The difference between these groups were not statistically significant (*P* >  0.05).

**Table 2:** Calculated OHD based on results from measurements on photographs

*Group*	*mean*	*sd*	*min*	*max*	*P-Value*
1 (dental advertisements)	1.39	0.30	0.69	1.75	> 0.05 ^n.s.^
2 (fashion advertisements)	1.34	0.35	0.81	2.10	> 0.05 ^n.s.^
3 (orthodontic advertisements)	1.23	0.41	0.00	1.84	> 0.05 ^n.s.^
4 (orthodontic textbooks)	0.62	0.18	0.33	0.98	< 0.001*

n.s., statistically not significant; *, *P* < 0.001; OHD, Occlusal height difference

The difference of the above groups, when compared to group 4 (orthodontic textbooks) however was statistically significant (*P* <  0.001). In group 4, the calculated average maxillary central-to-lateral OHD was 0.62 mm. This value is rather close to the 0.5 mm commonly prescribed protocols for bracket placement.

## Discussion

The results of this study revealed that maxillary central-to-lateral OHDs are usually greater than 1 mm for images in dental, fashion and orthodontic advertisements (1.39 mm, 1.34 mm and 1.23 mm). In contrast, the maxillary central-to-lateral incisor OHD was very close to 0.5 mm for orthodontic textbooks.

A number of investigators [7–10, 19, 20] found that the maxillary central-to-lateral OHD has an impact on perception of smile - aesthetics and this feature has been evaluated before. Ker et al. [7], using data from the Ohio state study, suggested a maxillary central-to-lateral incisal height difference of more than 0.5 mm was most aesthetic. In their computer-based investigation laypersons were asked to change the maxillary central-to-lateral incisal height difference in 0.1825 mm increments, until optimal aesthetics were achieved. Results exhibited values from 0 to 2.9 mm; negative values were disallowed.

Chan [21] found an ideal value of 1.4 mm OHD when changing the maxillary central-to-lateral incisal OHD in 0.18 mm increments. In an investigation by Machado et al. [9] images were digitally altered in order to create six different central incisor vertical positions in 0.5-mm increments. As a result, a maxillary central-to-lateral OHD of 1.5 mm was found most aesthetic. Bukhary et al. [10], also digitally manipulated dental configurations that were assessed. The length of the lateral incisor was altered in 0.5 mm increments to produce a total of five images with the lateral incisor 0.5 mm, 1 mm, 1.5 mm, 2 mm and 2.5 mm shorter than the adjacent central incisor. The authors found that an anterior dental arrangement with the maxillary lateral incisors 1.5 mm shorter than the adjacent central incisor was preferred by most assessors. In an investigation by King et al. [20], the vertical position of the maxillary lateral incisors was digitally morphed from a position of approximately 1.4 mm past the level of the central incisors to approximately 2.2 mm above the level of the central incisors. An animation of 43 frames in length were produced and assessed. A maxillary central-to-lateral incisor height difference of 0.5 mm was found to give best aesthetics. Brisman [22] used different non-digital dental setups and concluded that patients preferred an arrangement whereby the anterior teeth that are almost at the same horizontal plane; i.e. a maxillary central-to-lateral OHD of 0 mm. Interestingly, for studies where jurors preferred a maxillary central-to-lateral OHD of 1.5 mm, it made no difference whether aesthetics were changed by small increments or in 0.5 mm intervals [7, 10].

A number of factors need to be reviewed critically when assessing our investigation: In contrast to standardized clinical photography [23], acquisition of suitable photographic material from promotional photography, i.e. ‘genuine’ frontal views, was challenging. Variation of head position, can lead to parallax distortion and this can have an effect on linear measurements [24]. It is not questionable that advertisement photography is not standardized. Still, for best results professional photographers prefer focal lengths 85 mm in full-frame photography that show hardly any parallax distortion. This setting is preferred by most professionals and more important than a specific camera brand. Even an oblique shot with a portrait focal length with a full-frame camera would not have much influence on crown height perception. Wide angle lenses would create distortion if used close-up and are thus unsuitable for facial fashion photography. They would produce facial – and dental – features that could not possibly be regarded as attractive. It would appear nonsensical to use those pictures for measurement purposes. Therefore, it was the appearance of what advertising photography was willing to consider publishable was what we wanted to investigate; in other words, the looks rather than the exact measurement.

By calculating the differences (Δ) we found only minimal deviations from the values calculated using the 10.5 mm standard value. Although these deviations increased for larger OHDs, differences remained very small (please see Table 1) and hence have not compromised our results.

However, it cannot be denied that the inclination of the optical axis towards the motive (teeth in that case) leads to a certain amount of distortion on form of object lengthening or shortening, resulting in different perception of any given object. However, when it comes to reproduction of “beauty” as desired in advertisement and fashion photography, the shooter will instinctively cut out individual creativity through distortion but rather do everything for the outcome that suits the taste of the target group. Simply because the image has to be sold. Naturally, if the investigator is not the same person as the photographer, differences will remain, and an error must result. If related to OHD, this means that the measurement outcome will include certain errors. However, even with a slight amount of distortion, the difference between an OHD of 0.5 mm and 1.5 mm will still be detectable.

Our study assumed an average maxillary central incisor crown height of 10.5 mm [2, 18] and this was used as the standard value to calculate the maxillary central-to-lateral incisal height difference. This can lead to over - or underestimation of the calculated incisal height. However, the results for group 4 (orthodontic textbooks; mean 0.62 mm) suggest that the height difference could have been only (0.12 mm) overestimated; we also calculated results using different incisor heights and as per our calculation (Table 1), over - or underestimation of the maxillary central-to-lateral incisor OHD was very small and this could not have affected our results significantly. Image distortion due to non - standardized photographic technique is unlikely to have caused significant bias in our study; it is rather more likely that distortions cancelled each other out on average.

The results of our three advertisement groups are in agreement with those studies preferring a maxillary central-to-lateral incisal OHD of more than 1 mm [7, 9, 10, 21]. Not completely unexpected, the results for group 4 (orthodontic textbooks) showed a mean value of 0.62 mm for maxillary central-to-lateral OHD, which is close to the 0.5 mm used for standard protocols for bracket placement [5, 6].

Physical attractiveness is commonly used as an advertising tool [25]: “what is beautiful is good” [26]. A smiling face is known for its advertising appeal and the effect on customers has been evaluated before: the industry recommends use of smiling images over to non-smiling faces [27]. One would assume that for purposes of marketing, the more attractive the smile, the greater the effect on the customer. Several studies found maxillary central-to-lateral incisal height difference greater than 1 mm to be the most attractive [7, 9, 10, 21] and we assume that the advertisers’ selections of smiles were, consciously or sub - consciously influenced by this factor.

In order to avoid potential biased selection of the photographs, both lay persons as well as dental professionals were requested to contribute images. Consecutively, two investigators selected the photographs for the different study groups. Hence, a selection could be assumed due to personal preferences [28]. However, decision for inclusion or exclusion was made according to pre-set criteria, unlikely influencing our results.

Treatment planning in aesthetic dentistry usually begins at the maxillary central incisor area [29], and this applies to orthodontics [4]. To create a particular central-to-lateral incisor OHD, the orthodontist must either define the bracket position on the incisors as required or use artistic bends of the archwire. However, ‘white aesthetics’ are one of the considerations for achieving an aesthetic appearance: gingival tissues are generally known to follow individual tooth movements within reason [30–33] and that, in turn, will change the contour of the anterior gingiva, potentially necessitating minor gingivoplasty to achieve optimal aesthetics. Machado et al. [9], found that when the gingival margin of the central incisor matched the laterals it was rated most aesthetic. However laypersons are not able to differentiate gingival asymmetry of 0.5 to 1.5 mm between maxillary incisors [7, 29, 34, 35], suggesting that the vertical position of the incisors can be varied to some extent without causing dissatisfaction with orthodontic treatment.

The need for customized orthodontic (and potentially restorative) treatment applies to patients with missing maxillary lateral incisors, whose treatment plan may include space closure [36–38]. Re-arrangement of the maxillary incisor display may be required and to optimize aesthetics and deviation from commonly used bracket placement protocols should be considered, modifying the extent of central-to-lateral OHD. Bukhary et al. [10] found that hypodontia patients seem to prefer a maxillary central-to-lateral OHD of 1 mm. This value lies between the 0.5 mm, commonly used in standard protocols for bracket placement [5, 6] and values of greater than 1 mm, suggested by other investigators [9, 19, 21].

Although “ideal” values for OHD of about 1.5 mm were found in the literature, there seems to be a considerable diversity depending on individual preference. Ker et al. [7] showed that values ranging from 0 to 2.9 mm were acceptable suggesting: “Beauty is in the eye of the beholder” [28]. Patients’ opinions should be taken into account to avoid dissatisfaction with orthodontic treatment and OHD between central and lateral incisors should be considered at the treatment planning stage [39, 40].

## Conclusions

We recommend assessing patient opinion regarding maxillary central-to-lateral OHD in females at the treatment planning stage or at least during the final treatment stages including finishing and final adjustments. Modification of commonly used bracket placement protocols may be helpful achieving the desired aesthetic outcome.
